# Cold Shock-Induced Nanocomposite Polymer Packaging Maintains Postharvest Quality of Vegetable Soybeans

**DOI:** 10.3390/foods14234129

**Published:** 2025-12-02

**Authors:** Xiaogang Wang, Liangyi Zhao, Xiaohuan Liang, Yonghua Zheng, Peng Jin

**Affiliations:** College of Food Science and Technology, Nanjing Agricultural University, Nanjing 210095, China; wxgangw@163.com (X.W.); t2024063@njau.edu.cn (L.Z.); 15927680879@163.com (X.L.); zhengyh@njau.edu.cn (Y.Z.)

**Keywords:** *Glycine max* (L.) Merr., storage quality, preservation, cold shock, packaging performance

## Abstract

Vegetable soybean is a major crop in China with significant economic value. However, it is prone to yellowing and browning during postharvest storage, which reduces quality, marketability, and competitiveness. ‘Tongdou No. 6’ was used to evaluate postharvest quality preservation through combined cold shock treatment and nanocomposite polymer packaging. The results demonstrate that the combined treatment effectively slows the green-to-yellow color change by significantly reducing chroma a* value, weight loss, and chlorophyll degradation. Additionally, it markedly reduces the accumulation of malondialdehyde (MDA) and reactive oxygen species (H_2_O_2_ and O_2_·^−^), while decreasing the activities of polyphenol oxidase (PPO) and peroxidase (POD). The treatment also significantly enhanced the levels of antioxidant compounds, including ascorbic acid (AsA), total phenolics, and total flavonoids, and boosted the activities of key antioxidant enzymes—ascorbate peroxidase (APX), superoxide dismutase (SOD), phenylalanine ammonia-lyase (PAL), and catalase (CAT). Moreover, the combined application of cold shock and nanocomposite polymer packaging significantly enhanced the scavenging capacity against DPPH and hydroxyl (OH^−^) radicals. Overall, combining these two techniques effectively delayed senescence-related discoloration by activating the antioxidant system, regulating ROS metabolism, and reducing oxidative damage. This approach is highly effective in maintaining postharvest quality and offers a promising solution to the storage-induced deterioration of vegetable soybeans.

## 1. Introduction

Vegetable soybeans (*Glycine max* (L.) Merr.), members of the *Fabaceae* family, are annual herbaceous plants commonly referred to as edamame or green soybeans. They are harvested at an immature stage and characterized by their greenish-blue pods, which resemble peas in shape but possess a firmer texture. The pods are densely covered with fine trichomes, classifying them as a typical pod-consumed vegetable soybean variety [1,2]. With a cultivation and consumption history spanning thousands of years in China [3], vegetable soybeans hold considerable economic significance in domestic markets. Globally, they are widely appreciated for their rich nutritional composition, including carbohydrates, proteins, vitamins, minerals, and phytochemicals [4,5], and serve as a valuable source of dietary protein, fiber, and phytoestrogens [6]. However, postharvest, these vegetables are highly perishable and prone to quality issues such as yellowing, browning, dehydration, and shriveling. Prolonged storage accelerates senescence and spoilage. During storage and transport, they are susceptible to pathogens causing diseases like brown spot and black spot, which severely affect sensory quality, culinary value, and marketability [7].

The cold stimulation of fruits and vegetables using ice-water mixtures or cold air delays fruit decay and senescence by inhibiting ethylene and polyamine biosynthesis, reducing respiratory rate, and enhancing antioxidant enzyme activity. This treatment enhances the fruit’s intrinsic resistance, thereby effectively achieving preservation [8,9]. In sweet cherries, cold shock treatment has been shown to reduce weight loss; suppress malondialdehyde (MDA) accumulation; preserve firmness, color intensity, and total anthocyanin content; and decrease postharvest losses by modulating the expression of CSDPs genes [10]. For broccoli, this treatment results in improved quality parameters—such as firmness, titratable acidity, and water content—alongside increased levels of volatile compounds and enhanced sensory attributes. It also significantly increases the activities of antioxidant enzymes such as peroxidase (POD) and catalase (CAT), effectively prolonging shelf life [11]. In chili peppers, cold shock treatment markedly delays cumulative weight loss, maintains fruit color and firmness, and preserves overall quality during storage from 0 to 21 days by boosting antioxidant enzyme activities, including superoxide dismutase (SOD) and catalase (CAT) [12].

Traditional packaging increases environmental burden and may impair fruit respiration and transport quality. Developing biodegradable materials that match the respiratory needs of fruits and vegetables is therefore a key strategy for reducing environmental impact and maintaining postharvest quality [13]. The integration of nanofillers offers innovative opportunities to enhance material properties: titanium dioxide nanoparticles (TiO_2_ NPs) exhibit antibacterial, ultraviolet-absorbing, and photocatalytic characteristics, which improve the mechanical strength and gas barrier performance of packaging films [14,15]; silver nanoparticles are chemically stable and possess strong antimicrobial activity, making them among the most widely used nanoscale antimicrobial agents [16]; silica nanoparticles are recognized by the FDA as food additives, offering low cost, biocompatibility, and broad applicability in food packaging [17]. To improve nanoparticle dispersion, previous studies have used polyethylene (PE) as a matrix, incorporating silver, titanium dioxide, and silica nanoparticles along with attapulgite to fabricate nanocomposite bags. The large surface area of the nanoscale system enhances the loading capacity of bioactive compounds and improves film stability and mechanical strength [18]. In recent years, nanoparticle-reinforced packaging materials have been successfully applied to the preservation of blackberries [19], rice [20], and mushrooms [21], yielding favorable results that support their potential use to extend the shelf life of vegetable soybeans.

Based on current research on cold shock and nanocomposite polymer packaging, we speculate that combining the two may help to maintain edamame postharvest quality. In this study, cold shock treatment was applied to investigate its effects on antioxidant activity and reactive oxygen species (ROS) metabolism after harvest. The research aims to elucidate the efficacy of cold shock treatment in preserving vegetable soybeans and to provide theoretical foundations for its application in delaying senescence and browning, as well as prolonging storage duration.

## 2. Materials and Methods

### 2.1. Materials and Treatments

The vegetable soybean cultivar used in this study was ‘Tongdou No. 6’ (*Glycine max* (L.) Merr.), collected from Nantong City, Jiangsu Province. Harvesting occurred between growth stage R6 (seed filling: fully expanded, non-hardened seeds) and R7 (initial ripening: onset of mature pod coloration with bright green pigmentation). Immediately after harvest, samples were rigorously selected based on the following criteria: uniform pod size; consistent coloration without signs of yellowing or browning; absence of mechanical damage (e.g., crushing injuries or insect feeding holes); and no visible symptoms of pest infestation or disease infection. This screening process ensured initial homogeneity in the quality of the experimental material. Based on preliminary findings from this research group, the following five treatments were implemented: (1) Control (CK): vegetable soybeans were immersed in distilled water at room temperature (20 ± 1 °C) for 10 min. After ensuring the absence of visible water droplets, package the item in a polyethylene bag (PE). (2) Cold shock (CS) treatment group: soybeans were submerged in an ice-water mixture maintained at 0 ± 1 °C for 10 min. Following treatment, the beans were air-dried and packaged in polyethylene (PE) plastic bags with a thickness of 0.01 mm. (3) Standard packaging group: Vegetable soybeans were immersed in distilled water at room temperature (20 ± 1 °C) for 10 min. After ensuring the absence of visible water droplets, package the item in a polyethylene bag (PT). (4) Nanocomposite polymer packaging (NM): The preparation of nanocomposite polymer packaging materials was conducted following the method described by Fang et al. [21]. Vegetable soybean was enclosed in nano-preservation bags fabricated from a PE matrix incorporating composite additives, including silver nanoparticles, titanium dioxide nanoparticles, silica nanoparticles, and attapulgite (Nanjing Haitai Nano-Materials Co., Ltd., Nanjing, China). These bags were manufactured through a process of mixing, palletization, and blow molding to achieve a final thickness of 40 μm [21]. (5) Composite treatment group (CS&NM): soybeans were first immersed in an ice-water mixture at 0 ± 1 °C for 10 min, then air-dried and subsequently packaged in the aforementioned nano-preservation bags. Each treatment unit consisted of approximately 250 g of soybeans per bag, with three biological replicates assigned to each group. All samples were stored under controlled conditions at 20 ± 1 °C for a period of 10 days. Every two days, three replicate bags were sampled simultaneously for the assessment of key quality and physiological parameters.

### 2.2. Measurement of Browning Index, Weight Loss Rate, Vitamin C Content, Firmness, and Color Parameters in Vegetable Soybeans During Storage

The browning index was calculated according to the method described by Ye et al. [22]. For vegetable soybeans, the browning index was determined using the following formula:

Browning index (%) = [Σ (Browning degree of each pod grade × Number of pods in that grade)/3] × 100 (1)

The weight was measured using an electronic balance (HZ 602A, Cixi Hongzuan Weighing Instrument Equipment Co., Ltd., Cixi, China). The weight loss rate was calculated based on the method of Huang et al. [23], as follows:

Weight loss rate (%) = (Initial mass − Final mass)/Initial mass × 100(2)

Ascorbic acid (VC) content was determined following the procedure of Wang et al. [24]. Briefly, 1 g of pod tissue was weighed and homogenized in a mortar with 5% trichloroacetic acid (TCA) for extraction. After centrifugation (Centrifuge, 50121195, Manufactured by Thermo Electron LED GmbH, Osterode, Germany), the supernatant was collected. In a clean test tube, 1 mL of extract, 1 mL of 5% TCA, and 1 mL of anhydrous ethanol were added sequentially, followed by 0.5 mL of phosphoric acid (H_3_PO_4_), 1 mL of 0.5% o-phenanthroline solution, and 0.5 mL of 0.03% FeCI_3_ solution. The mixture was vortexed thoroughly and incubated in a water bath at 30 °C for 1 h. After incubation, the absorbance of the reaction solution was measured at 534 nm (UV-1800PC Ultraviolet Spectrophotometer, Shanghai Scientific Instrument Co., Ltd., Shanghai, China). A standard curve was constructed using ascorbic acid solutions of known concentrations, and VC content was expressed as mg 100 g^−1^.

Firmness was determined using the method of Dai et al [25]. Color changes in vegetable soybeans were evaluated using a chromameter (CR-400, Tokyo, Japan, Konica Minolta Ltd.) with random sampling. L, a*, and b* color coordinates were recorded for each sample.

### 2.3. Measurement of Chlorophyll, MDA, Total Phenolic Content, and Total Flavonoid Content in Vegetable Soybeans During Storage

The chlorophyll content was determined using the method described by Tian et al. [26], with some minor modifications. First, 1 g of pods was extracted using a 95% ethanol solution. The resulting supernatant was transferred to a 10 mL volumetric flask and topped up with 95% ethanol. Absorbance was measured at wavelengths of 649 nm and 665 nm. The results were expressed as μmol 100 g^−1^.

MDA was determined according to the method of Jing et al. [27]. Take 1 g of pods and extract the supernatant with a 10% TCA solution. In a test tube, combine 1 mL of the supernatant with 1 mL of the TBA solution. For the control group, substitute the enzyme solution with 10% TCA. Incubate in a boiling water bath for 30 min, allow to cool naturally, then centrifuge. Remove the supernatant and measure the absorbance values at 450 nm, 600 nm, and 532 nm as A_1_, A_2_, and A_3_, respectively. Results are expressed as nmol g^−1^.

Total phenols were determined according to the method of Jing et al. [27]. Extract 1 g of pods with a 70% ethanol solution and reserve the supernatant. In a fresh test tube, add 50 µL of the enzyme solution, 150 µL of distilled water, 1 mL of Folin–Ciocalteu reagent (99%), and 0.8 mL of sodium carbonate solution. Incubate at 30 °C for one hour, then measure the absorbance at 765 nm.

Total flavonoids were determined using the method described by Jing et al. [27]. Take 1 g of pods and extract the supernatant with a 70% ethanol solution for later use. In a fresh test tube, add 1 mL of the supernatant, 1.5 mL of 5% AlCl_3_ solution, and 1 mL of PBS buffer. Mix thoroughly, then incubate in a water bath at 30 °C for 15 min. Measure the absorbance at 430 nm.

### 2.4. Measurement of Hydrogen Peroxide (H_2_O_2_), Superoxide Anion (O_2_·^−^), Hydroxyl Radical (OH^−^) Scavenging Activities, and DPPH Radical Scavenging Capacity in Vegetable Soybeans During Storage

The H_2_O_2_ content and the rate of O_2_·^−^ generation were determined using a method adapted from Zhao et al. [28]. Briefly, 1 g of pod tissue was homogenized in pre-chilled acetone, followed by centrifugation to obtain the supernatant. Then, 1 mL of the supernatant was mixed with 0.2 mL of 20% titanium sulfate (TiSO_4_) solution, vortexed thoroughly, and allowed to react at room temperature for 1 h. Subsequently, 0.2 mL of concentrated ammonia solution was added to precipitate the complex. The precipitate was washed with cold acetone, centrifuged at 4 °C and 12,000× *g* for 10 min, and then dissolved in 3 mL of concentrated sulfuric acid (H_2_SO_4_). After a second centrifugation, the absorbance of the resulting supernatant was measured at 410 nm. A standard curve was constructed using known concentrations of H_2_O_2_, and H_2_O_2_ content was expressed as µmol g^−1^.

The O_2_·^−^ generation rate was determined according to the method of Wang et al. [29], with minor modifications. The reaction mixture included hydroxylamine hydrochloride and sulfamic acid (99%) under controlled conditions, and the formation of nitrite was monitored spectrophotometrically. Results were calculated based on the rate of O_2_·^−^ production and expressed as nmol min^−1^ g^−1^.

The hydroxyl radical (OH^−^) scavenging activity was assessed using the method described by Wang et al. [30] and expressed as a percentage. The scavenging capacity was calculated using the following formula:

OH^−^ scavenging activity (%) = [1 − (A − B)/A_0_] × 100 (3)

where A is the absorbance of the sample, B is the absorbance of the blank control (replacing reagent with solvent), and A_0_ is the absorbance of the control group (without extract).

The DPPH radical scavenging capacity was determined using a slightly modified version of the method of Wang et al. [30]. In brief, 1 g of pod sample was extracted with 5 mL of 50% ethanol, and the supernatant was collected as the test solution. For the assay, 0.1 mL of the supernatant was mixed with 1.9 mL of 0.1 mM DPPH solution in methanol. The mixture was incubated at 25 °C in the dark for 20 min. Absorbance was measured at 517 nm. A control group containing DPPH solution without extract was used as reference. The DPPH radical scavenging capacity was calculated as follows:

DPPH scavenging capacity (%) = [1 − (A − B)/A_0_] × 100 (4)

where A is the absorbance of the sample, B is the absorbance of the blank, and A_0_ is the absorbance of the control. Final results were reported as percentage inhibition.

### 2.5. Measurement of Reactive Oxygen Species-Related Enzyme Activities in Vegetable Soybeans During Storage

Polyphenol oxidase (PPO) activity was determined according to the method of Khoshalani et al. [31]. Briefly, 2 g of pod tissue was homogenized in 0.2 M phosphate buffer (pH 6.8) and centrifuged to obtain the supernatant. The change in absorbance of the reaction mixture was monitored spectrophotometrically at 410 nm for 1 min. One unit of PPO activity was defined as an increase of 0.01 in absorbance per minute per gram of fresh weight (OD min^−1^ g^−1^).

POD activity was measured following the method of Huang et al. [32], with minor modifications. The extraction procedure was identical to that used for PPO. The assay mixture contained guaiacol as substrate, and the increase in absorbance at 470 nm was recorded over a 1 min period. The result was expressed as OD min^−1^ g^−1^.

CAT activity was determined using the method described by Li et al. [33]. In brief, 1 g of pod sample was extracted with 0.05 M phosphate buffer (pH 7.0). In a clean test tube, 2.6 mL of the same buffer was mixed with 0.2 mL of 0.75% hydrogen peroxide (H_2_O_2_) solution, followed by the addition of 0.2 mL of enzyme extract. The decomposition of H_2_O_2_ was monitored by measuring the decrease in absorbance at 240 nm for 1 min. One unit of CAT activity was defined as a decrease of 0.01 in absorbance per minute per gram of fresh weight, and results were expressed as OD min^−1^ g^−1^.

Ascorbate peroxidase (APX) activity was assayed according to the method of Li et al. [33], using ascorbate as the substrate. The extraction procedure was the same as that for CAT. In a clean test tube, 2.6 mL of 0.05 mol L^−1^ phosphate-buffered saline (PBS, pH 7.0), 0.2 mL of enzyme extract, and 0.2 mL of 0.75% H_2_O_2_ solution were added sequentially. The reaction was initiated by adding ascorbic acid, and the decrease in absorbance at 290 nm was recorded over 1 min. APX activity was calculated based on the rate of ascorbate oxidation and expressed as OD min^−1^ g^−1^.

SOD activity was determined following the method of Li et al. [33] using the nitroblue tetrazolium (NBT) photoreduction assay under light exposure. SOD activity was defined as the amount of enzyme required to inhibit NBT reduction by 50%, and results were expressed as units per gram of fresh weight (OD min^−1^ g^−1^).

The activity of phenylalanine ammonia-lyase (PAL) was assayed according to the method described by Medda et al. [34].

### 2.6. Data Processing

Data were processed and visualized using Origin Pro 2021 (Origin Lab Corporation, Northampton, MA, USA), GraphPad Prism 9 (GraphPad Software Corporation, Boston, MA, USA), and Microsoft Excel. Statistical analysis was performed using GraphPad Prism 9. One-way analysis of variance (ANOVA) was used to compare the means of each treatment group, and Duncan’s multiple range test was conducted at a significance level of 0.05. Pearson’s correlation coefficient was calculated using Origin Pro 2021 to assess linear relationships among measured parameters. All experiments were conducted in triplicate, and results are presented as mean ± standard deviation (SD) of three independent replicates.

## 3. Results

### 3.1. Effects of Cold Shock Treatment on the Postharvest Physiological Quality of Vegetable Soybeans

**Figure 1:**
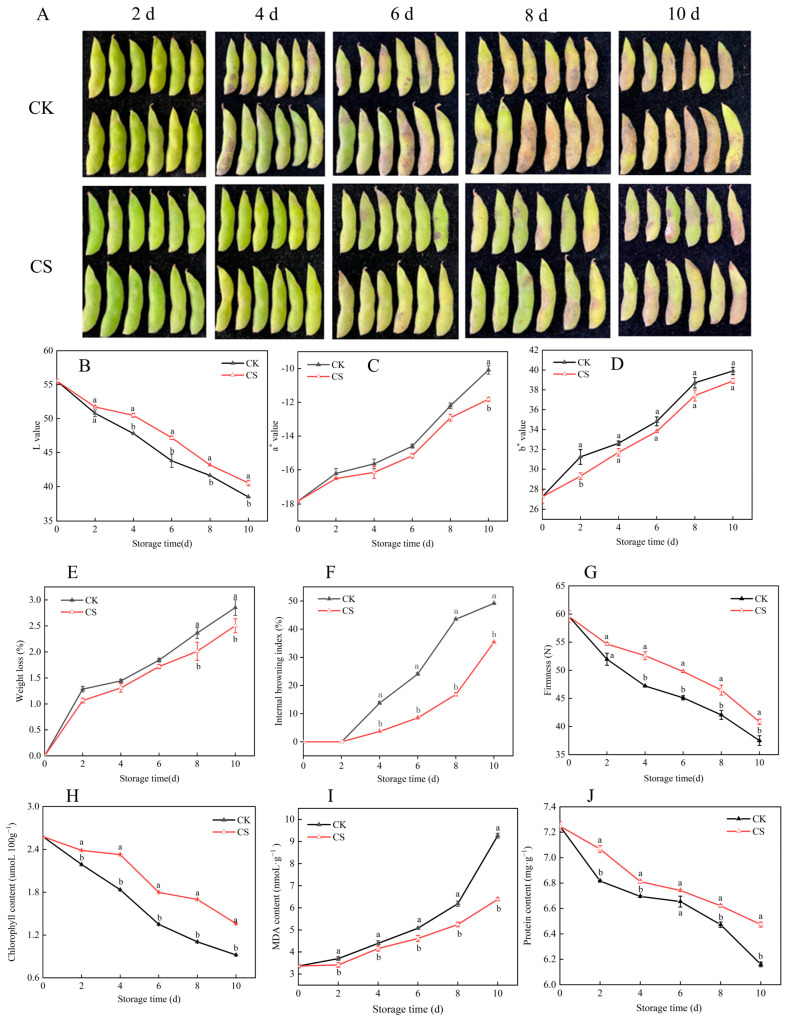
Effects of cold shock treatment on physical appearance (**A**), L value (**B**), a* value (**C**), b* value (**D**), weight loss rate (**E**), browning index (**F**), firmness (**G**), chlorophyll content (**H**), MDA (**I**), and protein content (**J**) in vegetable soybeans. Different lowercase letters within the same storage day denote statistically significant differences (*p* < 0.05), whereas identical letters indicate no significant difference.

External quality is a key indicator of fruit and vegetable freshness, serving as the most intuitive criterion for consumers and grading systems. In vegetable soybeans, pod appearance reflects freshness and edibility. Postharvest browning intensifies over time (Figure 1A), underscoring the need for effective preservation to maintain marketability. Compared to the control, cold shock treatment significantly delayed the decline in L value, with treated groups showing values 3.70% and 5.27% higher than control in later storage stages (Figure 1B), indicating a strong inhibition of color deterioration. Changes in chromaticity parameters a* and b* were substantially suppressed, indicating a better retention of greenness, reduced redness, and stable yellowness, which demonstrates the treatment’s effectiveness in preserving visual quality (Figure 1C,D). Water loss increased during storage: the control group exceeded 2% weight loss by day 8, while the cold-shocked group retained moisture more effectively (Figure 1E). Visible browning was delayed—localized discoloration appeared in the control by day 4, but the treated group remained intact until day 6. By day 8, image analysis showed a 61.39% reduction in browning severity with cold shock (Figure 1F), confirming its ability to suppress oxidative browning. Hardness declined progressively during storage, reflecting tissue softening and indirectly indicating cellular water loss and metabolic degradation (Figure 1G). To further investigate the physiological and biochemical impacts of cold shock, chlorophyll, MDA, and soluble protein contents were measured. Results showed progressive chlorophyll degradation and a significant decrease in soluble protein levels, indicating substantial nutrient loss during postharvest storage. Concurrently, increased cell membrane permeability led to sustained accumulation of MDA, a marker of lipid peroxidation. During the late storage period (days 6–8), the cold shock treatment group exhibited significantly slower deterioration rates for all three indicators compared to the control, with statistically significant differences (Figure 1H–J). In summary, cold shock treatment effectively retarded chlorophyll and protein degradation while suppressing MDA accumulation, thereby preserving both physiological activity and nutritional quality in postharvest vegetable soybeans. These findings underscore its potential as a practical strategy to delay senescence and enhance storage stability.

### 3.2. Effects of Cold Shock Treatment on Reactive Oxygen Species Metabolism and Antioxidant System in Vegetable Soybeans

**Figure 2:**
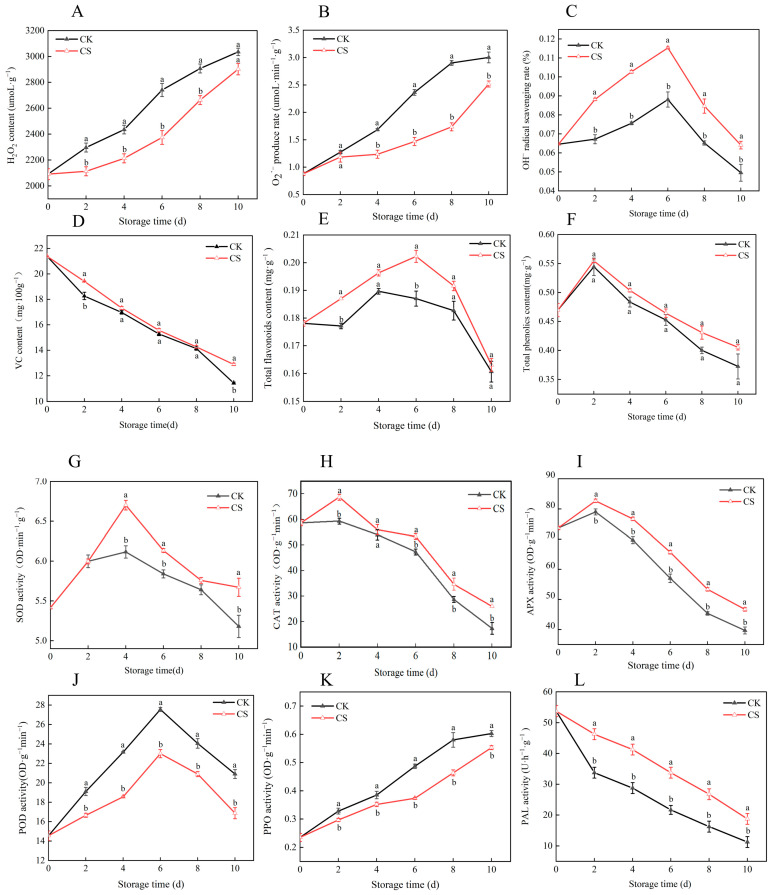
Effects of cold shock treatment on postharvest hydrogen peroxide content (**A**), O_2_·^−^ (**B**), OH^−^ (**C**), VC (**D**), total flavonoid content (**E**), total phenolic content (**F**), SOD (**G**), CAT (**H**), APX (**I**), POD (**J**), PPO (**K**), and PAL (**L**) in vegetable soybeans. Different lowercase letters within the same storage day denote statistically significant differences (*p* < 0.05), whereas identical letters indicate no significant difference.

H_2_O_2_ content and O_2_·^−^ production rate increased progressively during storage (Figure 2A,B). Although H_2_O_2_ levels were low, they showed continuous and marked accumulation, indicating sustained oxidative stress. Before storage, no significant difference in O_2_·^−^ production was observed between the CS and control groups. From mid- to late storage, however, the CS group had significantly lower O_2_·^−^ generation than the control (*p* < 0.05), demonstrating that cold shock effectively suppressed superoxide anion production. OH^−^ scavenging capacity followed a biphasic pattern—increasing initially and then decreasing—with a peak on day 6 (Figure 2C). The CS group maintained significantly higher scavenging activity throughout storage (*p* < 0.05), reaching a peak 1.3-fold higher than the control, reflecting enhanced antioxidant capacity. VC content declined continuously over time and was negatively correlated with storage duration. No significant differences were observed between groups in early or mid-storage, but, by day 10, the CS group retained 1.13 times more VC than the control (Figure 2D), indicating effective preservation of ascorbic acid in late storage. Total phenolic and flavonoid contents showed similar trends: initial increase followed by gradual decline (Figure 2E,F). The CS group maintained higher total phenolic content throughout storage (*p* < 0.05), suggesting that the treatment promotes phenolic compound biosynthesis or stabilization. Total flavonoids declined in the control from day 4, while the treated group maintained elevated levels until day 6, indicating delayed degradation; after day 6, the difference was no longer significant. SOD activity increased gradually early in storage, peaked on day 4, and then declined (Figure 2G). The CS group showed consistently higher SOD activity than the control, with the largest difference on day 4 (*p* < 0.05), indicating that cold shock effectively delayed the loss of enzymatic antioxidant capacity. CAT activity generally decreased over time, reaching its lowest level near the end of storage, with a transient peak on day 2 (Figure 2H). From mid- to late storage, CAT activity was significantly higher in the treated group than in the control (*p* < 0.05), suggesting that cold shock helps to preserve catalase stability. APX activity peaked on day 2 and then declined, with the treated group showing 4.64% higher activity than the control at this stage (Figure 2I), reflecting enhanced H_2_O_2_ scavenging capacity in the early post-treatment. POD activity initially increased and then declined (Figure 2J), with the cold-shocked group showing significantly lower peak activity than the control (*p* < 0.05). This suppression may improve cell wall integrity and reduce browning, thereby preserving visual quality. PPO activity, closely linked to oxidative browning, increased steadily in the control but remained significantly lower in the treated group throughout storage (Figure 2K, *p* < 0.05). Reduced PPO activity helps to mitigate oxidative damage, delay senescence, and inhibit pod discoloration, maintaining appearance and nutritional value. PAL, a key enzyme in the phenylpropanoid pathway, is essential for synthesizing phenolic and flavonoid antioxidants. PAL activity gradually declined after harvest (Figure 2L), reflecting reduced secondary metabolism; however, the decline was significantly slower in the treated group (*p* < 0.05), indicating that cold shock sustains metabolic activity and enhances intrinsic antioxidant capacity. In summary, cold shock enhances the postharvest antioxidant defense system in vegetable soybeans by coordinately regulating key antioxidant enzymes (SOD, CAT, APX, POD, PPO) and metabolic enzymes such as PAL. This synergistic modulation of ROS scavenging and secondary metabolic pathways effectively delays physiological aging and improves quality and storage stability.

### 3.3. Effects of Nanocomposite Polymer Packaging on Physiological and Nutritional Quality of Vegetable Soybeans During Postharvest Storage

**Figure 3:**
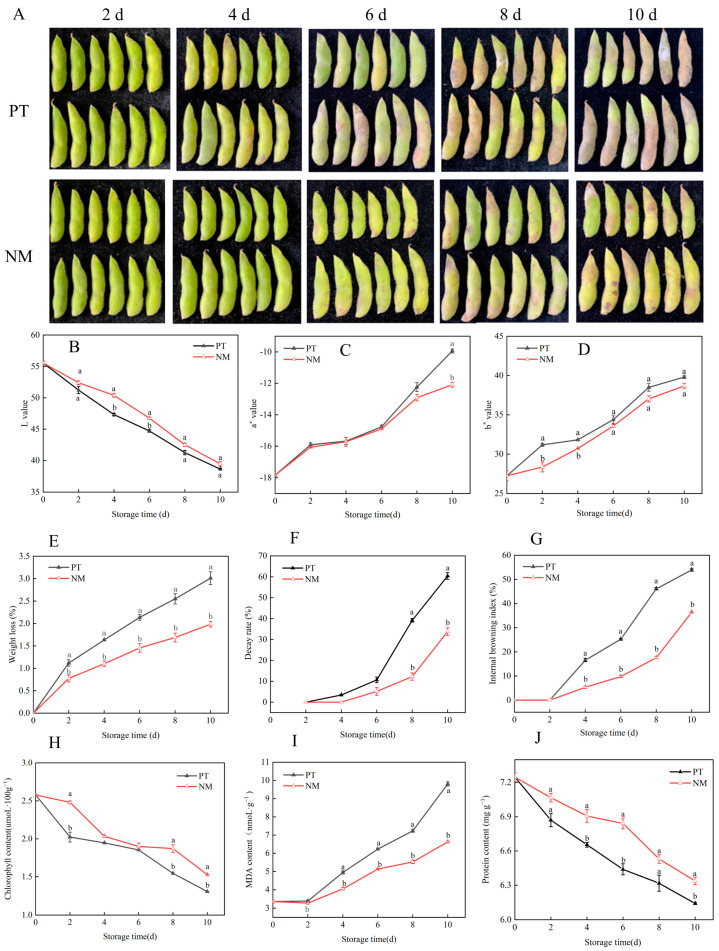
Effects of nanocomposite polymer packaging on physical appearance (**A**), L value (**B**), a* value (**C**), b* value (**D**), weight loss rate (**E**), decay rate (**F**), internal browning index (**G**), chlorophyll content (**H**), MDA (**I**), and protein content (**J**) in vegetable soybeans. Different lowercase letters within the same storage day denote statistically significant differences (*p* < 0.05), whereas identical letters indicate no significant difference.

To systematically evaluate the practical efficacy of cold shock treatment, this study compared the effects of conventional PE packaging and nanocomposite polymer packaging on vegetable soybean storage quality under controlled temperature conditions (20 ± 1 °C). Both methods partially mitigated pod browning; however, from day 6 onward, NM showed a significantly stronger inhibitory effect (Figure 3A), supporting the development of an integrated preservation strategy combining cold shock with NM. The browning index is a key indicator of postharvest quality in fruits and vegetables, reflecting color deterioration due to enzymatic browning (e.g., by polyphenol oxidase) or non-enzymatic reactions [27]. Over 10 days at 20 ± 1 °C, conventional PE packaging preserved visual quality during the first two days, making it suitable for short-term distribution; thereafter, browning progressed rapidly. In contrast, nanocomposite polymer packaging more effectively delayed browning onset and progression (Figure 3A,G), indicating greater potential for long-term storage. These changes were supported by color parameter trends: L decreased continuously, while a* and b* values gradually increased. The negative a* value indicates green pigmentation, and its rise reflects declining greenness and increasing redness, consistent with visible pod browning (Figure 3B–D). Weight loss increased progressively throughout storage, with the control group showing significantly higher rates than the nanocomposite polymer packaging group (*p* < 0.05), exceeding it by 44.50%, 48.17%, 46.75%, 51.15%, and 51.80% on days 2, 4, 6, 8, and 10—demonstrating nanocomposite polymer packaging’s superior moisture retention. However, rotting incidence in the NM group was significantly higher than in the PT group from day 8 to day 10 (*p* < 0.05), with increases of 219.59% and 78.70%, respectively, suggesting limitations in microbial inhibition and highlighting the need for integration with additional antimicrobial strategies (Figure 3E,F). Although both packaging types delayed browning, their overall performance was limited—by day 4, browning rates exceeded 5% in both groups (Figure 3G), indicating that packaging alone is insufficient for sustained quality control. Chlorophyll content declined continuously during storage, causing pronounced pod yellowing, with degradation trends closely matching changes in color parameters, particularly L and a* values (Figure 3H). Concurrently, cell membrane damage led to lipid peroxidation, shown by progressive MDA accumulation; meanwhile, soluble protein—a key nutritional component—declined steadily (Figure 3H–J), collectively reflecting the progressive deterioration of postharvest physiological metabolism and overall quality.

### 3.4. Effects of Nanocomposite Polymer Packaging on Reactive Oxygen Species and Antioxidant Quality in Vegetable Soybeans During Postharvest Storage

**Figure 4:**
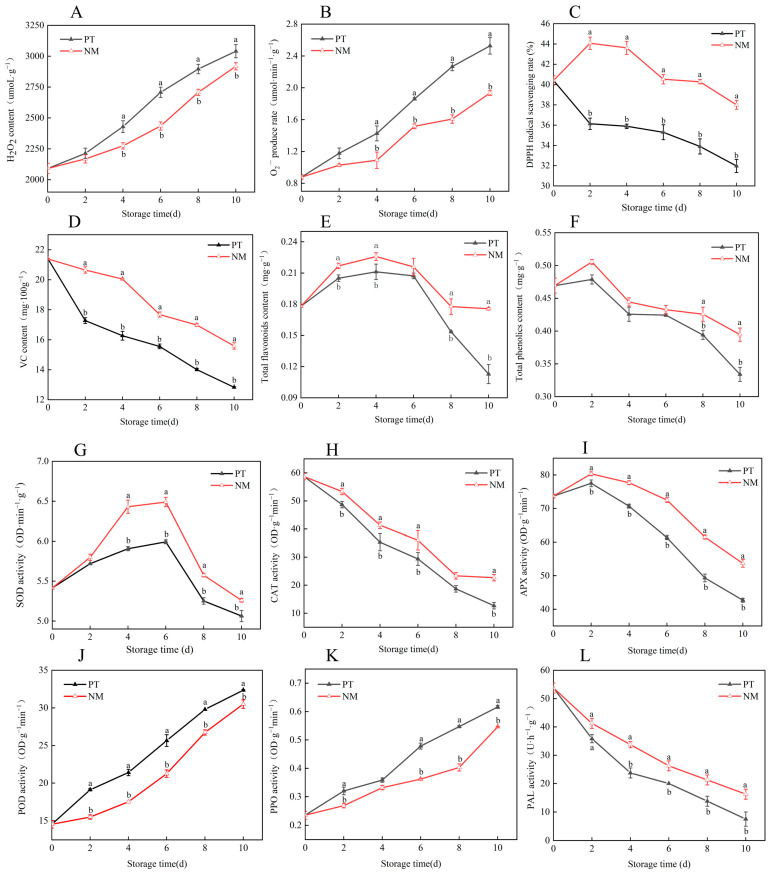
Effects of NM on postharvest H_2_O_2_ (**A**), O_2_·^−^ (**B**), DPPH (**C**), VC (**D**), total flavonoids (**E**), total phenols (**F**), SOD (**G**), CAT (**H**), APX (**I**), POD (**J**), PPO (**K**), and PAL (**L**) in vegetable soybeans. Different lowercase letters within the same storage day denote statistically significant differences (*p* < 0.05), whereas identical letters indicate no significant difference.

H_2_O_2_ content and O_2_·^−^ production rate in vegetable soybean pods gradually increased during storage at ambient temperature (20 ± 1 °C), peaking toward the end of the 10-day period (Figure 4A,B). Compared with the control (conventional PE packaging), nanocomposite polymer packaging significantly reduced both H_2_O_2_ accumulation and O_2_·^−^ generation throughout storage (*p* < 0.05), indicating the effective suppression of ROS buildup and oxidative stress damage. DPPH radical scavenging capacity in the nano-packaged group increased initially, peaked on day 2, and then gradually declined (Figure 4C). In contrast, the control group showed a continuous decrease, maintaining significantly lower antioxidant activity throughout storage (*p* < 0.05). On day 2, the DPPH scavenging rate in the treated group was approximately 1.2-fold higher than that of the control, and remained significantly elevated across all sampling points (*p* < 0.05), demonstrating that nano-packaging enhances the overall antioxidant capacity of vegetable soybeans. VC content declined progressively in both groups; however, NM significantly retarded VC degradation, thereby better preserving nutritional quality (Figure 4D). Total flavonoid and total phenolic contents followed a trend of initial increase followed by decline (Figure 4E,F). Total flavonoids peaked on day 4 before decreasing, with the NM consistently exhibiting higher levels than the control throughout storage—reaching approximately 1.5 times the control value by day 10. Total phenolics peaked on day 2 and then gradually declined; the NM group maintained higher levels at all time points, with differences becoming more pronounced in later storage stages (*p* < 0.05), suggesting that NM helps to stabilize key secondary metabolites. Antioxidant enzyme activities are shown in Figure 4G–I. Under ambient storage, SOD and APX activities initially increased and then declined, while CAT activity decreased continuously. The NM group showed significantly higher SOD, CAT, and APX activities than the control throughout storage (*p* < 0.05), indicating that NM preserves the functional integrity of the enzymatic antioxidant system and enhances the plant’s capacity to counteract oxidative stress. POD catalyzes phenolic compound oxidation and polymerization, contributing to tissue browning and senescence. POD activity increased steadily during storage but remained significantly lower in the NM group than in the control (*p* < 0.05), indicating an effective suppression of browning-related reactions (Figure 4J). PPO is a key driver of enzymatic browning; Figure 4K shows that NM significantly delayed the rise in PPO activity (*p* < 0.05), thereby reducing the rate and extent of pod discoloration. PAL, a regulatory enzyme in the phenylpropanoid pathway, reflects secondary metabolic activity. PAL activity declined continuously during storage (Figure 4L), indicating reduced postharvest metabolism and cellular function. The decline was significantly slower in the NM group, suggesting a partial mitigation of metabolic degradation. NM effectively reduced oxidative damage and quality loss in vegetable soybeans by modulating ROS production, enhancing free radical scavenging, maintaining antioxidant enzyme activities, and suppressing the overactivation of browning-related enzymes such as PPO and POD. These findings support the use of NM to extend the shelf life of fresh produce under ambient temperature conditions.

### 3.5. Effects of Cold Shock Combined with Nanocomposite Polymer Packaging on Physiological and Nutritional Quality of Vegetable Soybeans During Postharvest Storage

**Figure 5:**
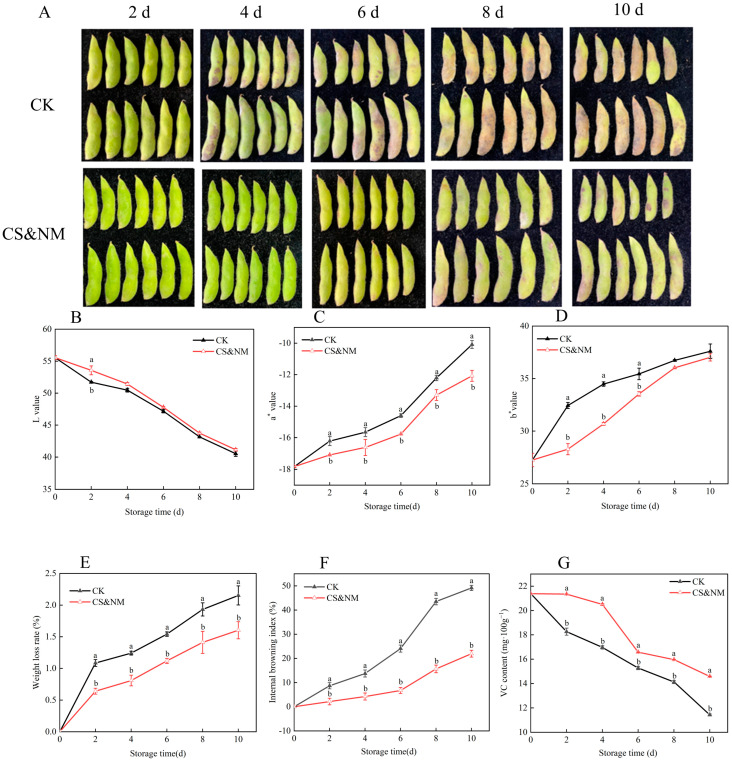
Effects of cold shock combined with NM treatment on the physical appearance (**A**), L value (**B**), a* value (**C**), b* value (**D**), weight loss rate (**E**), browning index (**F**), and VC (**G**) of vegetable soybeans. Different lowercase letters within the same storage day denote statistically significant differences (*p* < 0.05), whereas identical letters indicate no significant difference.

The changes in the visual quality of vegetable soybeans subjected to a combined cold shock and NM treatment during storage under controlled conditions (20 ± 1 °C, 80–90%) are shown in Figure 5A. The treated group maintained good color and structural integrity for the first 4 days, with only mild localized browning appearing by day 6. Compared to cold shock or NM alone, the combined treatment significantly reduced postharvest browning incidence (*p* < 0.05). These findings indicate that vegetable soybeans are highly susceptible to browning and water loss under ambient storage conditions. The CS&NM integration effectively delays these deterioration processes, thereby improving storage quality. Among color parameters, the L value showed a negative correlation with a* and b*, reflecting an inverse relationship between lightness loss and pod yellowing. Figure 5B–D shows color evolution during storage. The combined treatment markedly suppressed the increase in a* values—reducing them by 1.54% versus the control at the end of storage (*p* < 0.05)—demonstrating its effectiveness in preserving green color. Postharvest, vegetable soybeans are highly susceptible to water loss through transpiration. Weight loss increased progressively over time, but the combined treatment group had the lowest rate, with significant differences from the control (*p* < 0.05) (Figure 5E). This indicates that combining cold shock and NM reduces moisture loss by suppressing transpiration and respiration, thereby better maintaining pod turgor and visual quality. The browning index increased gradually during storage (Figure 5F). By day 4, it reached 2% in the treated group versus approximately 13% in the control (*p* < 0.05). By day 8, the control rose to 43%, while the treated group remained around 16%. From mid- to late storage, the combined treatment consistently showed lower browning indices than the control (*p* < 0.05), indicating an effective inhibition of oxidative browning, delayed senescence, and reduced spoilage risk. VC content declined continuously in both groups, but the control degraded faster. Statistical analysis showed a 47.6% reduction in VC in the control versus 28.6% in the treated group, with a significant difference (*p* < 0.05) (Figure 5G). Thus, the synergistic effect of cold shock and NM effectively mitigates ascorbic acid degradation, preserves nutritional value, delays aging, and enhances green retention. Since a* reflects greenness and VC content is a key indicator of senescence, their parallel trends further support the comprehensive benefits of this integrated approach in maintaining overall quality during storage.

### 3.6. Effects of Cold Shock Combined with Nanocomposite Polymer Packaging on Postharvest Reactive Oxygen Species Metabolism and Antioxidant Quality in Vegetable Soybeans

**Figure 6:**
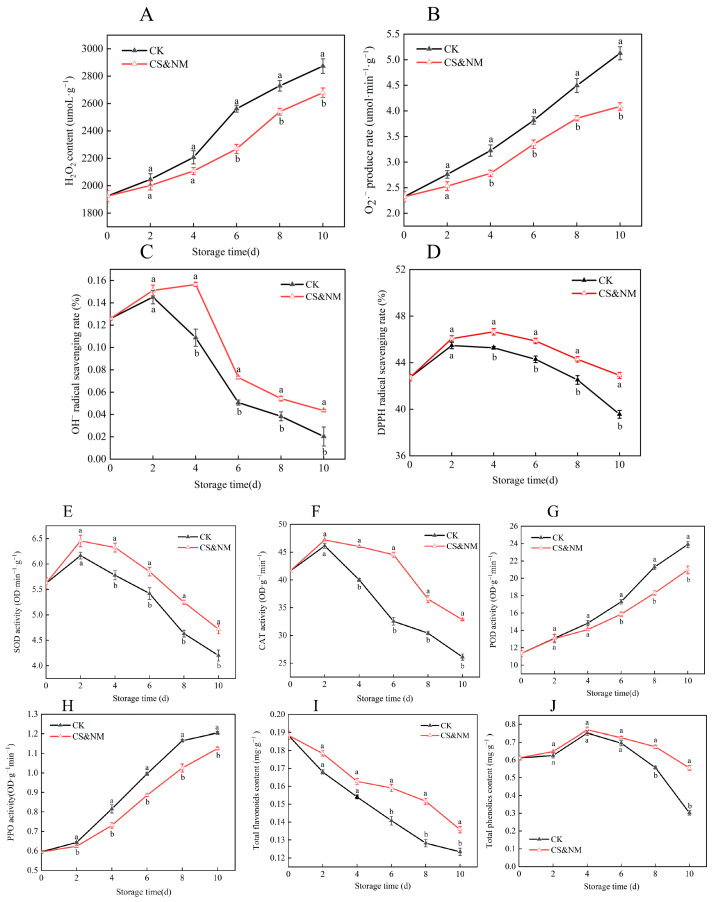
Effects of cold shock combined with NM on postharvest H_2_O_2_ (**A**), O_2_·^−^ (**B**), OH^−^ (**C**), DPPH (**D**), SOD activity (**E**), CAT (**F**), POD (**G**), PPO (**H**), total flavonoid content (**I**), and total phenolic content (**J**) in vegetable soybean. Different lowercase letters within the same storage day denote statistically significant differences (*p* < 0.05), whereas identical letters indicate no significant difference.

H_2_O_2_ content and O_2_·^−^ production rate in vegetable soybean pods increased continuously during storage, peaking toward the end of the period (Figure 6A,B). The composite-treated group showed no significant difference from the control during early storage; however, in mid-to-late stages, both H_2_O_2_ content and O_2_·^−^ production rate was significantly lower than in the control (*p* < 0.05). This indicates that combining cold shock with NM effectively suppresses excessive ROS accumulation during mid-to-late postharvest stages, thereby reducing oxidative stress damage. DPPH and hydroxyl radical scavenging capacity gradually increased during early storage and then declined after a certain point in both groups (Figure 6C,D). Throughout storage, the control group showed significantly lower radical scavenging capacity than the composite-treated group (*p* < 0.05), indicating superior antioxidant potential in the latter. The most pronounced decline in hydroxyl radical scavenging occurred on day 4, reflecting substantial stress on the antioxidant system at this stage. The composite-treated group declined more slowly, suggesting that CS&NM effectively delays the loss of DPPH and hydroxyl radical scavenging activities and maintains higher endogenous antioxidant activity. SOD and CAT activities increased initially and then gradually declined during storage, peaking on day 2 (Figure 6E,F). At that time, the composite-treated group showed SOD and CAT activities 4.65% and 2.31% higher than the control, respectively, and maintained higher levels throughout storage. These results indicate that the combined treatment enhances antioxidant defense responsiveness and delays enzyme activity decline, improving physiological stability in vegetable soybeans under oxidative stress. POD and PPO activities increased progressively under ambient storage conditions. These enzymes are key regulators of phenolic oxidation and enzymatic browning in fruits and vegetables, with elevated activities typically associated with tissue senescence (Figure 6G,H). Notably, PPO activity in the composite-treated group remained significantly lower than that in the control throughout storage (*p* < 0.05), while POD activity was markedly suppressed during the mid-to-late stages (*p* < 0.05). These findings suggest that the synergistic effect of CS&NM effectively delays enzymatic browning by inhibiting the overactivation of PPO and POD, thereby slowing the overall aging process. Total flavonoid content continuously declined during storage (Figure 6I). The composite-treated group consistently maintained higher levels than the control, with a significant difference on day 6 (*p* < 0.05), indicating that the treatment stabilizes secondary metabolites. Total phenolic content gradually increased during early storage, peaked on day 4, and then declined. In the late storage phase, the treated group had significantly higher total phenolic content than the control (*p* < 0.05), further confirming that the composite treatment helps to maintain phenolic accumulation and metabolic balance (Figure 6J). In summary, CS&NM synergistically delays postharvest physiological deterioration in vegetable soybeans through multiple mechanisms: suppressing ROS accumulation, enhancing free radical scavenging, maintaining antioxidant enzyme activities (e.g., SOD and CAT), reducing browning-related enzyme activities (e.g., PPO and POD), and delaying the degradation of total flavonoids and phenolics. This integrated approach thus significantly improves storage quality and physiological stability.

### 3.7. Correlation Analysis and Putative Regulatory Mechanisms

**Figure 7:**
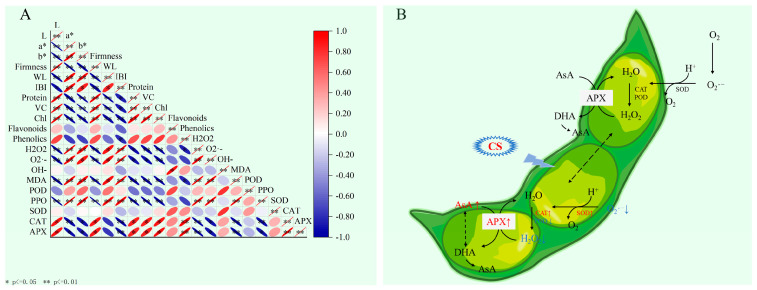
Heatmap illustrating the correlations among physiological quality attributes, ROS metabolism, and the antioxidant system in cold-treated vegetable soybean during storage, based on Pearson correlation analysis. WL: weight loss rate; IBI: browning index; ChI: chlorophyll content. Positive and negative correlations are represented by red and blue, respectively. A single asterisk denotes a significant correlation (*p* < 0.05), and double asterisks denote a highly significant correlation (*p* < 0.01) (**A**). Schematic representation of the potential regulatory mechanism underlying antioxidant defense in vegetable soybean (**B**).

This study used Pearson correlation analysis to assess the relationships between cold shock treatment and various physiological and quality parameters in vegetable soybeans (Figure 7A). The L value in the cold-shocked group was significantly positively correlated with firmness, protein content, VC, chlorophyll content, and CAT and APX activities (*p* < 0.05), with highly significant correlations (*p* < 0.01) for firmness, protein, VC, and chlorophyll content. L values also showed highly significant negative correlations (*p* < 0.01) with a* and b*, H_2_O_2_ content, O_2_·^−^, MDA content, and PPO activity, indicating that brightness retention is closely linked to the suppression of oxidative damage. The trends in a* and b* values were opposite to those of the L value, further confirming the synchrony between the loss of green color and increased yellowing or reddening during color deterioration (Figure 7A). Correlation analysis revealed that firmness was highly negatively correlated with weight loss rate, browning index, H_2_O_2_ content, O_2_·^−^ production rate, MDA content, and PPO activity (*p* < 0.01), indicating that tissue softening is strongly linked to water loss, oxidative stress, and enzymatic browning. In contrast, firmness was significantly positively correlated with CAT, APX, VC, protein, and chlorophyll content (*p* < 0.05), suggesting a synergistic relationship between the maintenance of cellular structural integrity, antioxidant capacity, and nutrient preservation. These findings suggest that cold shock treatment may effectively delay postharvest browning and color changes in vegetable soybeans by reducing weight loss, lowering the browning index, and suppressing the accumulation of oxidation-related compounds such as MDA and PPO. Furthermore, weight loss rate was significantly negatively correlated with protein, VC, chlorophyll, and CAT and APX activities (*p* < 0.05), while showing significant positive correlations with browning index, H_2_O_2_, O_2_·^−^, MDA, and PPO activity (*p* < 0.05), indicating that moisture loss is not merely a physical phenomenon but a comprehensive indicator of overall physiological deterioration. Chlorophyll content exhibited highly significant negative correlations (*p* < 0.01) with H_2_O_2_, O_2_·^−^, MDA, and PPO activity, and significant positive correlations (*p* < 0.05) with CAT and APX activity, demonstrating that cold shock helps to preserve chlorophyll stability by inhibiting ROS accumulation and key browning enzyme activities, thereby delaying pod yellowing and color degradation. In summary, Pearson correlation analysis revealed that cold shock effectively maintains postharvest physiological quality in vegetable soybean by suppressing H_2_O_2_, O_2_·^−^, and MDA levels, as well as PPO and POD activities. At the same time, this treatment enhances the activities of key antioxidant enzymes—CAT and APX—thereby strengthening endogenous ROS scavenging capacity and retarding quality deterioration. Cold shock treatment markedly delayed the decline in ascorbic acid (AsA) content while increasing the activities of SOD, CAT, and APX, thus enhancing the antioxidant defense system in vegetable soybean. This cascade of effects contributed to reduced generation rates of H_2_O_2_ and O_2_·^−^, helping to maintain intracellular ROS metabolic homeostasis, alleviate oxidative damage, and achieve the multidimensional regulation of postharvest quality (Figure 7B).

## 4. Discussion

Vegetable soybeans, a fresh-market legume, have commercial and edible qualities that are highly dependent on the postharvest condition. Harvested at the plump-seed stage (R6–R7), they exhibit high metabolic activity and pod surfaces densely covered with moisture-absorbing trichomes, creating a humid microenvironment that favors pathogen growth and increases rot incidence. Under ambient storage, high respiration and transpiration rates cause rapid dehydration and wilting, accelerating quality loss [35,36]. These traits make vegetable soybeans among the most difficult horticultural crops to preserve postharvest, underscoring the need for effective preservation technologies.

Cold shock treatment and NM technology, emerging approaches in postharvest preservation, effectively delay quality deterioration in multiple crop species by modulating antioxidant systems and maintaining cell membrane integrity. These effects provide a solid theoretical foundation and practical basis for investigating their application in vegetable soybeans. Extensive evidence indicates that cold shock and NM mitigate oxidative damage and maintain postharvest physiological stability through activation of endogenous antioxidant defense systems and suppression of excessive ROS accumulation in horticultural crops [37,38]. Concerning nanocomposite polymer packaging, Fang et al. applied nanocomposite treatment to enoki mushrooms and demonstrated that it significantly reduced H_2_O_2_, O_2_·^−^, and MDA levels [39]. This effectively alleviated lipid peroxidation-induced damage to cellular membranes, thereby preserving favorable sensory and nutritional qualities. The underlying mechanism is closely associated with the ability of nanomaterials to regulate membrane lipid peroxidation. In studies on cold acclimation, Wang et al. found that cold acclimation significantly delayed the decline in firmness and the accumulation of MDA during cucumber storage at 13 ± 2 °C, while effectively maintaining soluble solids content [40]. These results confirm the protective effect of cold acclimation on fruit and vegetable quality under low-temperature storage conditions. Further research by Mi et al. demonstrated that a 90 min cold shock treatment effectively suppressed postharvest water loss in peppers, preserved flavor quality and fruit firmness, and enhanced antioxidant capacity by increasing the activities of antioxidant enzymes such as SOD and CAT, thereby extending shelf life [12]. This highlights the need to optimize parameters to achieve maximal preservation efficacy. Overall, studies show that both cold shock and NM can significantly delay postharvest senescence in horticultural crops by modulating ROS metabolic pathways and maintaining cell membrane structural integrity, providing strong preliminary evidence for the composite preservation strategy explored in this study. To reduce postharvest deterioration in vegetable soybeans, we combined cold shock treatment (10 min immersion in 0 ± 1 °C ice water) with NM (a 40 μm polyethylene-based film containing nano-silver, nano-TiO_2_, nano-SiO_2_, and attapulgite). The treated group showed significantly higher SOD, CAT, and POD enzyme activities than the control. Concurrently, it delayed the decline in DPPH radical scavenging activity and hydroxyl radical scavenging capacity, while significantly suppressing the accumulation of H_2_O_2_ and O_2_·^−^. These findings are consistent with previously reported synergistic effects of cold shock and NM, further confirming that the combined treatment enhances ROS scavenging capacity through a synergistic activation of the endogenous antioxidant enzyme system in vegetable soybeans, thereby preventing oxidative damage to cellular membranes caused by excessive ROS. The reduced ROS accumulation directly lowered lipid peroxidation levels, thus preserving the structural and functional integrity of cell membranes. This not only minimized the leakage of intracellular nutrients but also reduced the risk of microbial infection, ultimately contributing to disease mitigation, quality retention, and extended storage duration [41]. Concerning MDA, a key indicator of membrane damage, the nanocomposite polymer packaging-treated groups—including the composite treatment group—significantly inhibited MDA accumulation during storage. As a major byproduct of lipid peroxidation, MDA levels directly reflect the extent of cellular membrane injury [42,43]. MDA concentrations lead to membrane disruption and increased permeability, triggering metabolic imbalances and tissue senescence. On the other hand, silver nanoparticles release Ag^+^ ions under humid conditions, exerting antibacterial and bactericidal effects that reduce the microbial degradation of cell membranes [16]. This explains why the weight loss rate in the treated group remained significantly lower than that in the control group (*p* < 0.05), accompanied by higher chlorophyll content and reduced browning. Maintained hydration helps preserve cellular turgor pressure, while chlorophyll retention sustains the fresh green appearance of pods. Collectively, these effects contribute to enhanced commercial quality and marketability of vegetable soybeans.

The decline in the postharvest nutritional quality of vegetable soybeans is mainly caused by abiotic stress-induced respiration and nutrient loss. Among the affected bioactive compounds, total phenolics and flavonoids are not only key nutrients but also core components of the non-enzymatic antioxidant system, with levels strongly linked to overall antioxidant capacity [42,43,44,45,46]. In this study, the NM group—including the composite treatment—maintained higher levels of these compounds and showed significantly greater DPPH and hydroxyl radical scavenging activities than the control. These results align with those reported by Xuan et al. [47] on loquats, Jia et al. on strawberries [48], and Li et al. on apples [49], which collectively demonstrate that NM or coating treatments enhance fruit antioxidant capacity by modulating secondary metabolic pathways to promote antioxidant synthesis or delay degradation. Combined with the observed increase in antioxidant enzyme activities, the cold shock–NM composite treatment established a more robust antioxidant defense network through synergistic interaction between the enzymatic antioxidant system (e.g., SOD, CAT) and the non-enzymatic antioxidant system (e.g., phenolics, flavonoids). This dual mechanism not only delays ROS-induced cellular damage more effectively but also minimizes the oxidative degradation of phenolic compounds, thereby preserving nutritional integrity. Notably, postharvest browning in vegetable soybeans represents a major factor compromising commercial quality, primarily mediated by elevated PPO and POD activities. These enzymes catalyze oxidation reactions between phenolic substrates and ROS, leading to the formation of brown polymers and disruption of internal tissue structure [50,51,52]. During the early storage phase, mild abiotic stress from cold shock and NM activated the antioxidant system, resulting in enhanced enzyme activity. As storage progressed, however, natural senescence led to a progressive decline in metabolic capacity and enzyme function. Despite this trend, the treated groups consistently maintained significantly higher enzyme activities than the control group across all time points (*p* < 0.05). This sustained enhancement underscores the prolonged activating effect of the combined cold shock and NM treatment on the endogenous antioxidant system of vegetable soybeans, contributing to improved oxidative stability and extended shelf life. Maintaining the steady-state structure of the dual mechanism is an effective approach to preserving postharvest quality in horticultural crops [53,54,55]. However, the inherent complexity of mechanistic research limits the accurate assessment of actual shelf life to some extent, thereby contributing to significantly elevated pre-sale costs. Consequently, the development of rapid and efficient preservatives is critically important and represents a key requirement for current industrialization efforts. Future studies should systematically investigate postharvest preservation technologies for horticultural crops from multiple dimensions.

## 5. Conclusions

This study systematically evaluated the effect of cold shock treatment on reducing browning and maintaining the postharvest quality of vegetable soybean ‘Tongdou 6’ during storage at 20 ± 1 °C. Cold shock significantly suppressed browning, enhanced stress tolerance, and extended shelf life, indicating its potential for ambient-temperature supply chains. The mechanism is linked to the upregulation of ROS-scavenging enzymes—especially SOD and CAT—which reduce reactive oxygen species accumulation, alleviate oxidative stress, and preserve cell membrane integrity. Intact membranes minimize electrolyte leakage and prevent degradative enzymes from interacting with substrates, thereby inhibiting enzymatic browning. Additionally, cold shock promoted the accumulation of key antioxidants, such as total phenolics and flavonoids, likely through increased antioxidant enzyme activity and the activation of secondary metabolism. These changes collectively strengthen the plant’s antioxidant defenses and delay senescence, providing dual protection for postharvest quality.

However, the mechanisms by which cold shock regulates NM-induced antioxidant pathways are not fully understood, and the molecular basis of their synergy—especially regarding gene expression and signaling crosstalk—remains unclear. Future studies should combine transcriptomics and metabolomics, and validate results across different cultivars and conditions to strengthen the theory and support industrial application.

## Data Availability

The original contributions presented in the study are included in the article, further inquiries can be directed to the corresponding author.
